# Characterizations of MWCNTs Nanofluids on the Effect of Surface Oxidative Treatments

**DOI:** 10.3390/nano12071071

**Published:** 2022-03-24

**Authors:** Norshafiqah Mohd Saidi, Mohd Nurazzi Norizan, Norli Abdullah, Nurjahirah Janudin, Noor Azilah Mohd Kasim, Mohd Junaedy Osman, Imran Syakir Mohamad

**Affiliations:** 1 Centre for Defence Foundation Studies, Universiti Pertahanan Nasional Malaysia, Kem Sungai Besi, Kuala Lumpur 57000, Malaysia; shafiqah.msaidi@yahoo.com (N.M.S.); mohd.nurazzi@gmail.com (M.N.N.); nurjahirahjanudin@gmail.com (N.J.); azilah@upnm.edu.my (N.A.M.K.); junaedy@upnm.edu.my (M.J.O.); 2 Research Centre for Chemical Defence, Universiti Pertahanan Nasional Malaysia, Kem Perdana Sungai Besi, Kuala Lumpur 57000, Malaysia; 3 Faculty of Mechanical Engineering, Universiti Teknikal Malaysia Melaka, Hang Tuah Jaya, Durian Tunggal 76100, Malaysia; imran@utem.edu.my

**Keywords:** CNT, MWCNTs, nanofluids, polyvinylpyrrolidone, PVP, thermal conductivity

## Abstract

In this study, multi-walled carbon nanotubes (MWCNTs) were chemically modified using three acid treatment methods to introduce the surface oxygen functional group (SOFG). The presence of SOFG on the MWCNTs has been characterized by Fourier Transform Infrared (FTIR) spectroscopy. Morphology, structural and thermal properties were performed using Field Emission Scanning Electron Microscopy (FESEM), Raman spectroscopy, and Thermogravimetric analysis (TGA), respectively. The result shows that the modification with acid treatment significantly affects the degree of defects and surface group functionality of surface oxidized MWCNTs from method B. The preparation of nanofluids using MWCNTs produced from method B (MWCNT-MB) was prepared using two different parameters: with and without polyvinylpyrrolidone (PVP) as surfactant. The experiment was conducted by setting variable carbon particle concentration from 0.1 wt.% to 1.0 wt.%, and the amount of PVP is 10% of carbon particles at different temperatures (6 °C, 25 °C, 40 °C). Based on visual observation, the dispersion of carbon particles was enhanced by the presence of PVP as the stabilizing agent. The thermal conductivity performance of nanofluids revealed that the surface oxidized MWCNTs with PVP show enhanced thermal conductivity compared to the nanofluid containing MWCNTs without PVP. The improvement contributes to this in terms of stability and homogenization of nanoparticles. Hence the improved distribution of MWCNTs in water-based media improves thermal conductivity. These promising properties of MWCNTs in water-based fluids would enable the nanofluids to be used in heat transfer fluid and cooling applications.

## 1. Introduction

The increasing demand for heat absorption, coupled with a tiny size component in any heat application, pushes researchers to look for a more efficient heat transfer system. Thermal conductivity is a characteristic of the rate at which heat can be transmitted through a material. This property plays a considerable role wherever heat transfer should be enhanced, and thus, it is the most-studied property of nanofluids. The addition of nanoparticles increases the thermal conductivity of host fluids [1]. The efficient heat transfer system helps to remove the heat generated and keep the devices under controlled operating conditions. However, the actual improvement in heat transfer efficiency may depend on a few parameters such as temperature, volume, type of surfactant, and duration for the synthesis process of the nanoparticles. At the same time, thermal conductivity is directly and positively proportional to the temperature and nanoparticle concentration [2]. Research indicates that substituting traditional air-cooling systems (i.e., ethylene glycol, oil, water, etc.) with liquid cooling ones is a promising solution to satisfy the increasing demand for high heat flux removal, maintaining low-temperature gradients, and wall overheating. Additionally, liquid cooling with high thermal conductivity is demanded in many industrial fields to develop heat transfer fluids. Commonly, nanoparticles have higher thermal conductivities than traditional fluids by one to three orders of magnitude [3]. Thus, a suspension of nanoparticles in a fluid will contribute to a higher thermal conductivity than pure fluids. The mixture then can be called a nanofluid. For example, Barbés et al. (2014) studied the thermal conductivities of CuO–water and CuO–Ethylene Glycol nanofluids [4]. It is observed that, for a given volume fraction and temperature, the thermal conductivity of CuO–water nanofluid is higher. In addition, Agarwal et al. (2016) studied the thermal conductivities of CuO nanofluids, mixing CuO nanoparticles with water, ethylene glycol, and engine oil [5]. The observations are consistent with those of Barbés et al. (2014), who found that the thermal conductivity of CuO-water nanofluid is higher than that of CuO-ethylene glycol nanofluid. The results show that CuO-engine oil nanofluid has the lowest thermal conductivity. Studies have shown that the change in the thermal conductivity of nanofluids with different base fluids is related to the different thermal conductivity of the base fluid and the strength of the interaction between the CuO nanoparticles and the base fluid.

According to Godson et al. (2010), at 70 °C, the maximum and minimum enhancement in thermal conductivity of Ag/water nanofluids were 27 and 80% for the Ag volume fractions of 0.3 and 0.9%, respectively [6]. In another study, Ranjbarzadeh et al. (2019) found that by adding 3% SiO_2_ nanoparticles into the water at 55 °C, the thermal conductivity of the host fluid increased 38.2% [7]. Another study on metal oxide is on the heat transfer from the potential of Al_2_O_3_ with the base fluid was the mixture of water and mono ethylene glycol as a car radiator coolant found the heat transfer performance of radiator is enhanced by using nanofluids compared to the conventional coolant [8]. In nanofluid with the lowest 0.2% volume fraction, a 30% rise in heat transfer is observed. The estimation of reduction in the frontal area of the radiator if base fluid is replaced by nanofluid is performed, which will make a lighter cooling system, produce less drag and save the fuel cost. According to both the experimental and theoretical analyses conducted by Soylu et al. (2019), the heat transfer performance of the nanofluids is dependent on the type of nanoparticle (TiO_2,_ Ag, and Cu) used in the fluids [9]. It was concluded that the precipitation potential of nanoparticles that could not disperse well in the nanofluid prevented the use of this heat transfer potential. The study conducted for the 0.1% and 0.3% Ag-doped nanofluids based on the performance factor clearly showed that the pressure loss due to increased viscosity after the higher volume of nanoparticles was negligible considering the increase in heat transfer. In conclusion, it can be said that doping with Ag would be beneficial for nanofluids, and the improvement increases with increasing doping ratio.

Other than that, nanofluids containing a suspension of carbon-based nanoparticles such as carbon nanotubes (CNTs), carbon nanofibers (CNFs), and graphite in the range 1 to 100 nm in a homogeneous fluid have attracted researchers’ attention [10,11,12,13]. The usage of nanoparticles could have enhanced heat transfer capabilities encountered with larger particles (size in the range of millimetres to micrometres). However, the large particles could clog the device’s channels. Saidur et al. (2011), Das et al. (2006), and Wang and Mujundar (2007) have reported the capabilities of nanofluids as heat transfer. They agreed that an accurate parameter in the synthesis of carbon particles must be considered to produce an efficient nanofluid. In most cases, carbon particles in nanofluid are advantageous to the heat transfer point of view only when the conditions are unfavorable for traditional thermal fluids [14]. Moreover, the use of carbon particles in nanofluid makes it possible to design a compact size radiator that also reduces the weight of the system, reduces drag, and saves fuel cost. Various types of nanomaterials and base fluids with high thermal conductivity were used as nanofluids, where nanocarbon materials such as MWCNTs have shown remarkable higher thermal conductivity than metallic solid materials such as aluminium, copper, and silver. Meanwhile, the water-based fluid has higher thermal conductivity than other base fluids. Many researchers have proved that the thermal conductivity of nanofluids increases with the thermal conductivity of the base fluid. 

In other applications, MWCNT also has been utilized in the denitrification process. The study of MWCNTs on the denitrification performance by *Alcaligenes* sp. TB has been studied [15], which showed that 30 mg/L MWCNTs increased NO_3_^−^-N removal efficiency from 84% to 100% and decreased the NO_2_^−^-N and N_2_O accumulation efficiency by 36% and 17.5%, respectively. Nitrite reductase and nitrous oxide reductase activities were further increased by 19.5% and 7.5%, respectively. The mechanism demonstrated that electron generation (NADH yield) and electron transport system activity increased by 14.5% and 104%, respectively [16]. Though, it is not clear whether the slight inhibitory effect of Pd-Fe on *Alcaligenes* sp. TB could be avoided by loading on MWCNTs with strong biocompatibility, whether the loading of Pd-Fe could affect the redox characteristics of MWCNTs, and whether it could further enhance the electron transport activity during the denitrification process of *Alcaligenes* sp. TB. Another study utilizing MWCNTs in the denitrification process is by Wang et al. (2021) [17]. A nanocomposite (Pd-Fe/MWCNTs) with chemical reduction ability and redox mediator properties was prepared to explore the effect on the denitrification performance of *Alcaligenes* sp. TB. The use of 30 mg/L Pd-Fe/MWCNTs have shown an excellent promotion on denitrification (completely TN removal at 36 h). Meanwhile, enzyme activity results indicated that the generation of NO_2_^−^-N, NH_4_^+^-N by Pd-Fe/MWCNTs led to the occurrence of short-cut denitrification by increasing 203.9% the nitrite reductase activity. Furthermore, electrochemical results and index correlation analysis confirmed that the electron exchange capacity (1.401 mmol eg^−1^) of Pd-Fe/MWCNTs was positively related to nitrite reductase activity, indicating its crucial role in electron transport activity (0.46 μg O_2_/(protein·min) at 24 h) during denitrification process by Pd-Fe/MWCNTs played a role of chemical reductant and redox mediator.

A sensitive material based on Pd-decorated MWCNT was used to fabricate a resistive sensor and investigated for the evaluation of its performance in the speciation of hydrogen isotopes (^1^H and ^2^H). The results obtained for the gas sensor revealed that the developed sensitive material can be used for isotopic identification of ^1^H and ^2^H and confirmed the existing reaction mechanism of Pd–H complex formation. Although the sensorial material has shown a good performance of hydrogen isotope sensing and quantification, further testing is foreseen on a higher number of sensors than considered in this study, variation of loading ratios for Pd onto MWCNTs in order to establish the most suitable one, tests for assessing the chemical stability, the reproducibility and mechanical stress in extreme conditions which either high or low temperatures [18]. Other than that, MWCNTs also has been widely used as sensing materials for toxic gases [19], as structural reinforcements [20,21], and have improved the thermal stability and electrical conductivity of the polymer nanocomposites [22,23].

CNTs have always been the focus of attention due to their exceptional power and excellent mechanical, electrical, thermal, and magnetic properties [24]. It has received significant attention due to its large intrinsic thermal conductivity and low density compared to metals or metal oxide nanoparticles [25]. The major weakness of carbon nanotubes is that they are hydrophobic and thus difficult to be dispersed in most solvent matrixes. Dispersion is one of the main factors that influence the properties of nanocomposites. Due to the presence of attractive forces (Van der Waals forces), nanomaterials tend to agglomerate from their polarizable, extended-electron systems [26]. To overcome this limitation, CNT surfaces are often tailored using two surface modifications: covalent [27,28] and non-covalent modification [29]. The covalent surface modification involves directly incorporating a new element or organic functionalities into the CNT sidewalls. A covalent surface modification effectively improves the adhesion and chemical properties of the composites, but it exhibits the disadvantage of disturbing the graphitic structure of CNT sidewalls [30].

The dispersion of carbon particles in liquid/fluids can be challenging. This is due to the strong van der Waal forces between carbon surfaces. For example, MWCNTs are hydrophobic, making them hard to disperse in water solution under any ambient conditions [31]. Based on the published data to date, many researchers have started to use dispersant agents such as surfactants to prevent the sedimentation or agglomeration of nanoparticles in which the surfactant produces an efficient coating and induces electrostatic or steric repulsions that could counterbalance van der Waals attractions [32]. However, the usage of surfactants in larger amounts has a few drawbacks, such as it can cause contamination in heat transfer media. Moreover, surfactant molecules will react and attach to the surface of carbon particles which could increase the thermal resistance between the carbon particles and the base liquid. This could reduce the thermal conductivity of carbon particles due to an increment in the density and viscosity of nanofluid [33,34,35]. As we know, heating and cooling are routine processes in heat transfer media. However, the presence of surfactants can lead to the production of foams, especially during heating [36]. All these factors could hinder the performance of heat transfer media.

Therefore, it is crucial to synthesize carbon particles either without or with a small amount of surfactant to achieve outstanding thermal performance, which could help extend its application. In this work, surface modification via acid treatment has been used. This treatment introduced SOFG on carbon walls and significantly affected the degree of defect. SOFG can increase the interaction between carbon particles and water molecules via hydrogen bonding. As a result, it has increased the hydrophilicity of the carbon particles and prevented their settlement in water over time. The thermal conductivity of carbon-based nanofluids is best associated with functionalized nanofluids. In addition, using PVP in a small amount of 10% of carbon particles as a dispersant agent in nanofluid will improve the stability or dispersibility of nanoparticles in fluids. Moreover, many researchers have less explored the combination of SOFG based nanocarbon combined with PVP as a surfactant in nanofluid preparation.

Hence in this study, commercial MWCNTs were chemically modified using three different acid treatment methods to introduce SOFG. The presence of SOFG on the carbon materials has been characterized by FTIR spectroscopy. Morphological, structural, and thermal properties analyses were performed using FESEM, Raman spectroscopy, and TGA. Based on the results obtained, modification with acid treatment presented a significant effect on both the degree of defects and surface group functionality of all samples. The procedure for the preparation of nanofluids was followed by using two different parameters: with and without surfactant, PVP. the nanofluids performance has been characterized through the observation on stability and dispersion analysis of nanofluid and thermal conductivity of nanofluid.

## 2. Experimental

### 2.1. Materials

In this study, the commercial MWCNTs used is of a 95% purity, the specific surface area is 200 m^2^/g, outside and has an inside diameter with 10 to 20 and 5 to 10 nm, respectively, these were purchased from the Nanostructured & Amorphous Materials, Inc., Texas, United States of America. Whereas the two types of acid used for the chemical oxidation modification of nanocarbon were concentrated sulphuric acid (H_2_SO_4_, 98%), nitric acid (HNO_3_, 65%), sodium nitrate (NaNO_3_), potassium permanganate (KMnO_4_), and hydrogen peroxide (H_2_O_2_) purchased from Merck kGaA, Darmstadt, Germany. All the chemicals were of analytical reagent grades and used as received without further purifications. The non-ionic surfactant used, polyvinyl pyrrolidone (PVP), was purchased from R & M Chemicals, Essex, United Kingdom.

### 2.2. Surface Oxidative Treatments of MWCNTs

The modification of MWCNTs with acid treatment was employed in this research due to its significant effect on the degree of defects and surface functionality that may provide enhanced thermal properties [37]. Three different acid treatment methods were used. For the first method (Method A), in a three-neck round bottom flask, 2 g of commercial MWCNTs were immersed in a mixture of 98% concentrated sulfuric acid (H_2_SO_4_) and 65% nitric acid (HNO_3_) in a ratio of 3:1 (*v*/*v*). The mixture was placed in an ultrasonication water bath (Fisher Scientific, MA, USA, FB 15057, frequency: 50 to 60 Hz) at 313 K for 30 min to disentangle the nanocarbon from aggregation and agglomeration in order to provide a functional anchoring group on the carbon surface. After that, it underwent reflux treatment at 423 K for 180 min. Finally, the modified nanocarbon powder was washed with deionized water several times and filtered through a 0.43 µm cellulose nitrile membrane filter until it reached pH 7. The powder was dried in a vacuum oven at a temperature of 323 K for 24 h prior to use. The second (Method B) and the third experiment (Method C) had the same process as Method A, though the reflux process was eliminated. However, different times of ultrasonication were employed with 2 h for Method B, and 6 h for Method C at 343 K. Table 1 shows the summary of three (3) acid treatment methods.

### 2.3. Preparation and Formulation of MWCNTs Nanofluid

Based on characterization analysis data on surface oxidative treatment of MWCNTs nanoparticles, MWCNTs nanoparticles prepared using method B (MWCNT-MB) were used for the preparation of nanofluid.

In this experiment, 40 mL of ultrapure water was used as the base fluid for the preparation of nanofluids in a glass container. The ultrapure water was produced using a Milli-Q Direct Water Purification System with specific characteristics (µS/cm, T, TOC, CFU/mL, Eu/mL). Two types of nanofluids were prepared: without PVP and with PVP. The amount of MWCNTs used in the formulation was ranged from 0.1 weight percent (wt.%) to 1.0 wt.% in the interval of 0.1. In comparison, the PVP used for the nanofluid formulation is of a 10% concentration of MWCNTs nanoparticles. The formulation ratio of water, MWCNTs, with and without PVP is presented in Table 2 and Table 3. In order to obtain the total volume of all materials, a formula can be used to calculate the synthesis of nanofluid, which can be referred to as Equation (1), where V is volume (mil), *m* is mass (g), and *ρ* is density. The pure base fluid (standard) used is ultrapure water.

(1) $$V=\frac{m}{\rho }\;(\mathrm{mL})$$

The two-step process was used to disperse the MWCNTs nanoparticles in a water-based nanofluid. This physical dispersion can be accomplished by using D1000 Handheld Homogenizer from Benchmark Scientific Inc., Sayreville, United States of America and Fisher Scientific FB 15057 Ultrasonic Water Bath. The samples were mixed perfectly by homogenizing for five minutes using a D1000 Handheld Homogenizer at a rotation speed of 10,000 rpm. This homogenization is essential to ensure that the carbon particles in water-based nanofluids are uniformly dispersed. Then, the samples underwent an ultrasonication process at room temperature (25 °C) for 30 min at a frequency of 60 Hz with the generation of output power being 240 W. This process is important to avoid the agglomeration of the nanofluid by breaking the Van der Wall interaction between particles by applying ultrasonic waves. The nanofluid samples were homogenized once again for 5 min at the rotation speed of 10,000 rpm.

### 2.4. Characterizations 

The structural analysis was characterized using a Perkin Elmer Frontier spectrometer FTIR (BX FTIR (Perkin Elmer, Beaconsfield, England)) with a scanning range of 500 to 4500 cm^−1^ and 16 scan numbers. Due to the black characteristics of carbon particles of MWCNTs, a pre-preparation of samples needs to be performed using a very low concentration of carbon particles in a potassium bromide (kBr) powder with a ratio of 1:9 and pressed into a disc shape. While, the structural analysis through Raman spectroscopic measurement was carried out according to Nurazzi et al. (2021) [22], using a Renishaw inVia Reflex Confocal Micro Raman System (Renishaw plc, Wotton-under-Edge, Gloucestershire, UK). The HPNIR laser for sample excitation was set at a wavelength of 787 nm, 1200 mm^−1^ gratings, and the magnification is 100× objective lens. The surface morphological analysis and the diameter size distribution of the commercial and after oxidation of nanocarbon were characterized using a FESEM JEOL 7600F, Carl Zeiss Gemini FESEM 500 (Carl Zeiss AG, Jena, Germany). Prior to the analysis, samples were initially coated with platinum (Pt) for 30 s in order to avoid charging. The diameter of the MWCNTs nanoparticles was measured using ImageJ software with 50 points for each sample. The thermal stability analysis, TGA was conducted using a TGA-DSC HT 3 analyzer (Mettler Toledo, Selangor, Malaysia) from a temperature of 35 to 1000 °C at a heating rate of 5 °C/min under a nitrogen gas flow atmosphere to study the stability of prepared nanocomposites.

For the MWCNTs nanofluids, the stability of MWCNTs nanofluids with and without PVP was observed through the sedimentation photograph capturing method. The clustering or sedimentation of the MWCNTs nanoparticles in the nanofluids can be investigated using this method. In this study, the nanofluid samples were left for 100 h after undergoing the ultrasonication process. Then, the sedimentation of the carbon particles can be observed from the photographic image, which is the sedimentation was captured using a camera [38]. KD2 Pro Analyzed (Decagon, Pullman, WA, USA) with water-based fluid, KS-1 (1.3 mm diameter × 60 mm long), and a specific accuracy of 5% was used to investigate the thermal conductivity of the MWCNTs nanofluid samples. The KD2 Pro Analyzed is calibrated with a standard glycerin solution at room temperature each time before measurement. In this study, the thermal conductivity was measured at three different temperatures, which are 6 °C, 25 °C, and 40 °C. Each sample was placed in a vessel, and the needle sensor was inserted into the sample. Before starting the thermal conductivity measurement, both sample and the needle sensor were maintained for 30 min at the required temperature. Once temperature stability was achieved, three measurements were recorded with 15 min intervals between each measurement. The experiment was conducted in a quiet environment to reduce fluctuation.

## 3. Results and Discussion

### 3.1. FTIR Analysis

**Figure 1:**
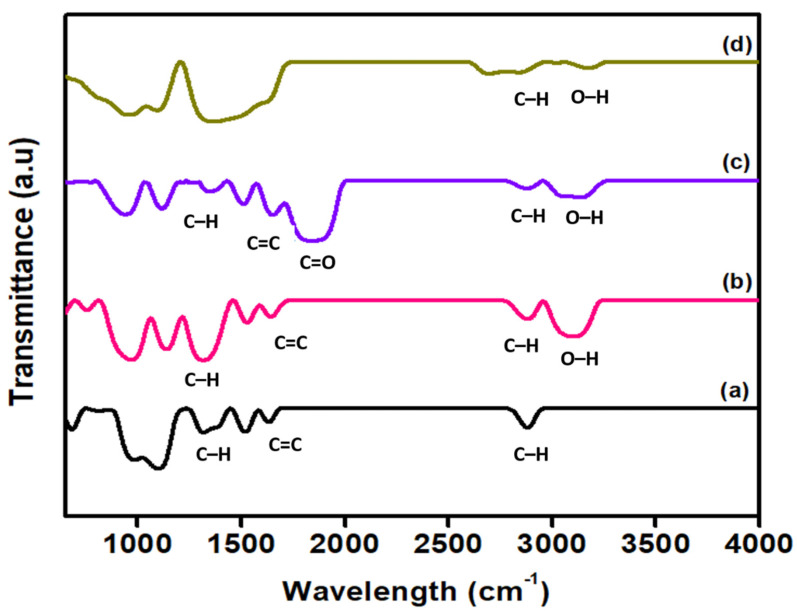
FTIR spectra of commercial and surface oxidized MWCNTs: (**a**) commercial MWCNTs (**b**); MWCNT-MA (**c**); MWCNT-MB and (**d**) MWCNT-MC.

The introduction of surface functional groups after the different surface oxidative treatments such as carboxyl (-COOH), hydroxyl (-C-OH), and carbonyl (-C=O) onto the MWCNT surface walls are presented in Figure 1. FTIR of commercial MWCNTs in Figure 1a shows several predominant peaks at 2880 cm^−1^ and in a range between 1600 cm^−1^ and 1350 cm^−1^. The peak at 2880 cm^−1^ and 1350 cm^−1^ region correspond to C-H asymmetric and symmetric stretching vibrational, respectively. The peak around 1600 cm^−1^ corresponds to C=C aromatic ring stretching related to MWCNT in nature [39,40]. Kouklin et al. (2004) studied MWCNTs of 60 nm diameter and reported spectrally uniform IR spectrum with a 100 meV bandgap and IR-active peaks at 1725 cm^−1^ (COOH groups), 1584 cm^−1^ (G band), 1200 cm^−1^ (D band), and several peaks in the range of 3000 cm^−1^ range that were attributed to CHx groups [41]. However, the broad peaks associated with the O-H stretch of the hydroxyl group around 3500 cm^−1^ are absent from the sample. This might be due to the purification process by the manufacturer. After treatment with the acid mixture H_2_SO_4_/HNO_3_, a strong peak appears in the wavenumber ranging from 2950 cm^−1^ to 3230 cm^−1^. This peak is assigned to the vibration of O-H bonds and can be related to hydroxyl groups and carboxyl groups. This region is not only assigned to the hydroxyl and carboxyl groups but also can be due to the moisture in the KBr powder, which is not completely eliminated [40]. The peak of the O-H bond shown in Figure 1d is very weak due to the structure of the MWCNTs being highly damaged. Additionally, a new peak appears (Figure 1c) in the range 1673 cm^−1^ to 1970 cm^−1^, which is attributed to the C=O groups in the different environments, which are carboxylic acid, ketone, or quinone [42]. This result clearly indicates that the functional group is successfully attached to the surface of MWCNTs.

### 3.2. Raman Spectroscopy Analysis

**Figure 2:**
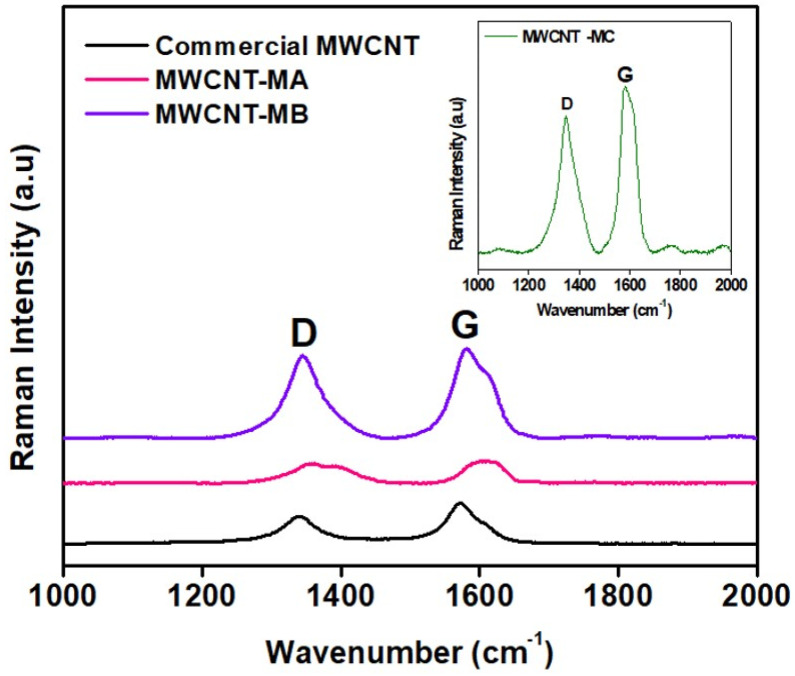
Raman spectrum of commercial and surface oxidized MWCNTs.

Figure 2 presents the Raman spectrum of surface oxidized MWCNTs. In the high-frequency region of the spectrum, two bands were observed showing the characteristics of CNTs; these bands point to the graphitic band (G band) and the disorder or defects of the structure, named D-band. The D-band is caused by a double resonance scattering due to the presence of structural defects, while the G-band originated from tangential in-plane vibration of graphitic carbon atoms [43]. All samples were observed to have peaks near 1350 cm^−1^ and 1590 cm^−1^, which were designated to the D and G band, respectively. MWCNT-MC has a higher intensity D-band peak compared to the other three samples. This reflected that the MWCNT-MC sample had high defects on its MWCNTs wall, which was in agreement with the FESEM images. The G-band has slightly shifted up field by about 5 to 6 cm^−1^ compared to commercial MWCNTs. It slightly shifted due to the presence of the functional group on the surface of MWCNTs. The values of intensity ratio I_D_/I_G_ for the commercial and surface oxidized MWCNTs are summarized in Table 4. The higher intensity ratio of the nanomaterials indicates higher defects due to the success of the oxidation process onto the MWCNTs sidewall. The value of the I_D_/I_G_ ratio is 0.75 for the commercial MWCNTs. The surface oxidation process that increases the ratio the most, and thus introduces the most defect in the MWCNTs, is the surface oxidized by Method B (MWCNT-MB) (I_D_/I_G_ = 0.91). Comparing the ratio I_D_/I_G_ values of surface oxidation and commercial MWCNTs, it is observed that the ratio values increase as expected after surface oxidation. The oxidation of MWCNTs breaks some of its bonds and inserts chemical groups that can be interpreted as defects in the structure. Comparing types of surface oxidation, all processes show a relevant change in the intensity of band D. These results indicate certain insertion of defects and/or breaks on the structure of MWCNTs.

### 3.3. TGA Properties

**Figure 3:**
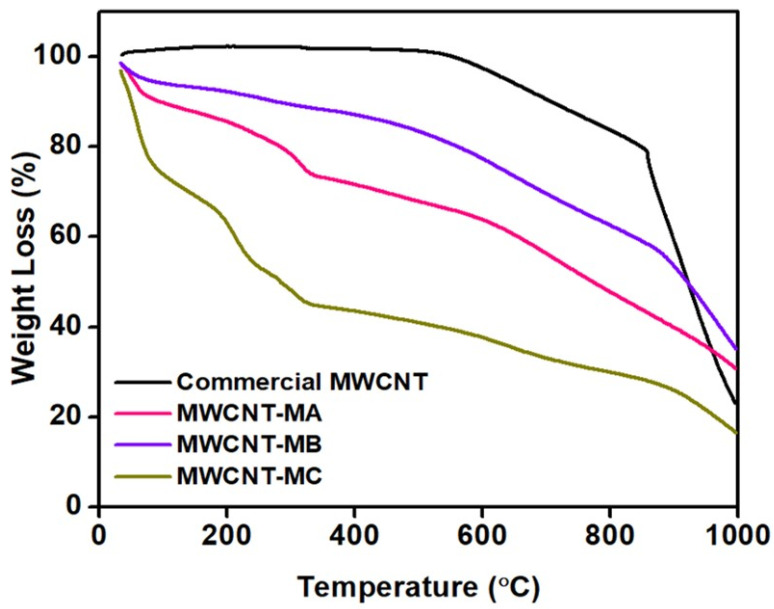
TGA thermogram of commercial MWCNTs, MWCNT-MA, MWCNT-MB and MWCNT-MC.

Figure 3 depicts the TGA results for commercial MWCNTs and the other three types of surface oxidized MWCNTs. As seen from the data, the commercial MWCNTs showed no change in weight loss until the temperature reached 530 °C. The commercial MWCNTs started to decompose beyond 530 °C and completely decomposed beyond 850 °C. The drastic weight loss occurring above 530 °C in the commercial MWCNTs is related to the degradation of disordered or amorphous carbon and other metal impurities, as reported in previous investigations [37,44]. By the TGA thermogram of all samples, a slight weight loss at 100 °C could be observed. This weight loss is assigned to the weight loss of moisture absorbed in MWCNTs. The initial weight loss or initial degradation of surface oxidized MWCNTs from 250 °C to 600 °C is assigned to the weight loss of the COOH group attached to the surface of MWCNTs. The weight loss is about 3.25%, 4.08%, and 8.43% for MWCNT-MA, MWCNT-MB, and MWCNT-MC, respectively. Around 600 °C to 800 °C, this weight loss is related to the oxidation of MWCNTs due to the decomposition of the COOH group, which is the carboxyl group releasing oxygen into the chamber of the TGA system [45]. Results obtained on TGA analysis demonstrate a good agreement with the results of the Raman spectroscopy. The indication of the maximum temperature of decomposition of surface oxidized MWCNTs is supported by the increasing ratio I_D_/I_G_ values for the oxidized MWCNTs, which is due to the oxidant being successfully attached at the active sites of MWCNTs [46] and improving the thermal stability of the MWCNTs samples.

### 3.4. FESEM Analysis

**Figure 4:**
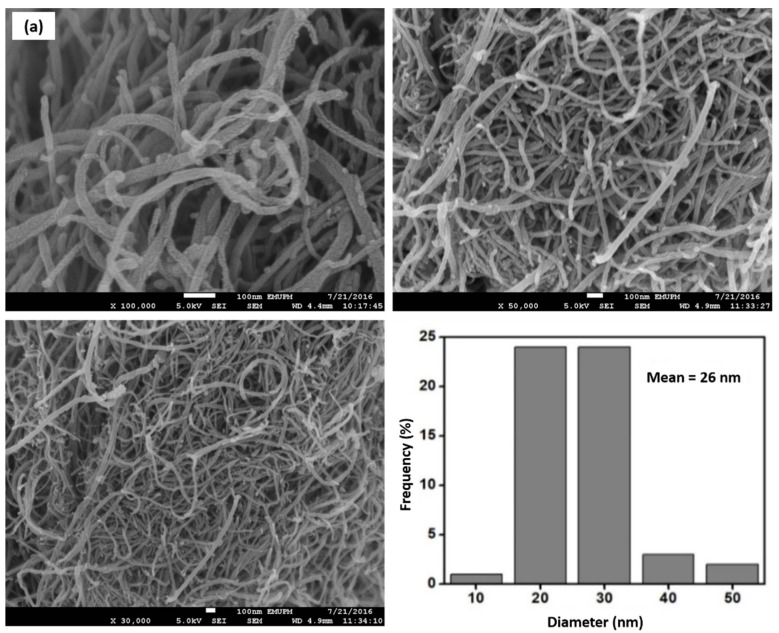

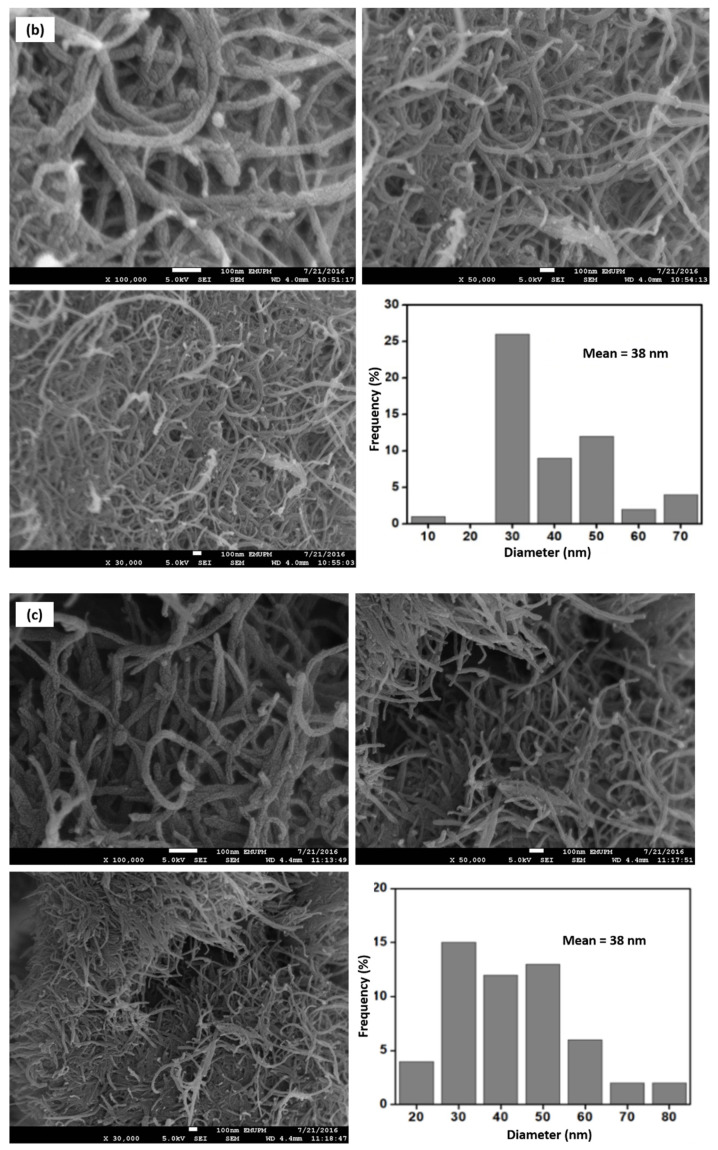

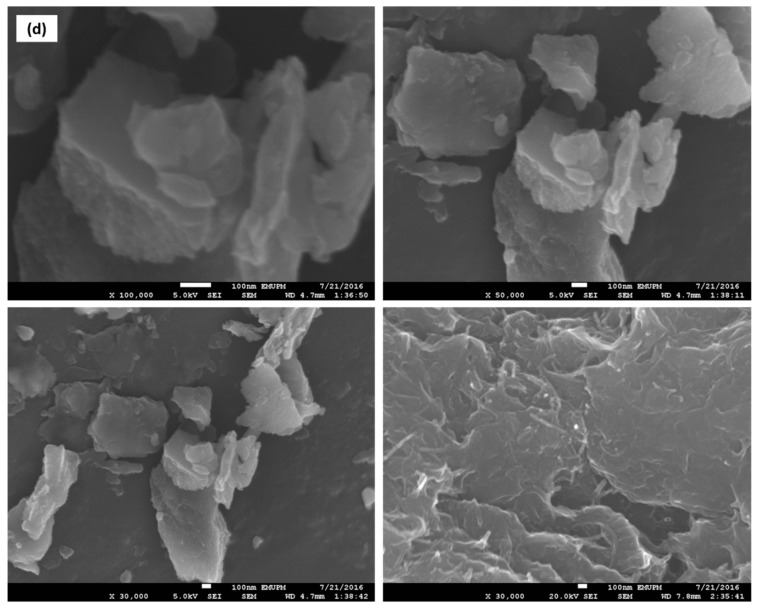
FESEM images of (**a**) commercial MWCNTs, (**b**) MWCNT-MA, (**c**) MWCNT-MB and (**d**) MWCNT-MC with different resolution and diameter distribution histogram.

Figure 4a–d represent the FESEM images of commercial MWCNTs, MWCNT-MA, MWCNT-MB, and MWCNT-MC, and their corresponding diameter size distribution histogram different resolution. The commercial MWCNTs in Figure 4a clearly show that the walls’ surface is smooth with random growth, inhomogeneous diameter distribution, and is highly entangled. The van der Waals forces attraction between the tubes cause the formation of bundles and entanglements. The diameter distribution of commercial MWCNTs is in the range of 10 nm to 50 nm, with a mean diameter of 26 nm. The arrangement of MWCNTs became debundled and disentangled following chemical and physical treatment using method A and Method B. Some areas show that the tubes are exfoliated and become curled. Additionally, the diameter distribution of MWCNTs increased, and the mean diameter of MWCNT-MA and MWCNT-MB are in the range of 38 nm and 42 nm, respectively. However, the stronger power of acid treatment causes several etchings of the graphic surface, leading to tubes with disordered sites and damage on the walls. This is indicative of defect creation along their walls due to the addition of new functional groups such as carboxylic and hydroxyl groups leading to the presence of disordered sites on the MWCNTs surface. The statement above is entirely consistent with our Raman spectroscopy results presented below. However, no shortening of tube length was observed for MWCNTs sample treated using method A. This indicates that the MWCNTs are strong enough to be unaffected by the concentrated acid solution used during treatment. Lui et al. (1998) used the acid mixture H_2_SO_4_/HNO_3_ on the highly tangled long of MWCNTs into short besides open-ended tubes and thus allowed any functional groups at the open end to be attached [47]. On the other hand, surface treatment using method C reveals a densely packed structure of MWCNT-MC. This is because the tubular structure of nanotubes has been significantly destructed and changed. This was caused due to the MWCNTs being exposed to high temperatures for a more extended reaction time (6 h). Therefore, as a result, the CNT diameter was unable to be determined. The overall SEM images showed that the acid treatment process performed at different period affect the bundling arrangement of nanotubes.

### 3.5. Stability and Dispersion Analysis of Nanofluids (MWCNT-MB)

**Figure 5:**
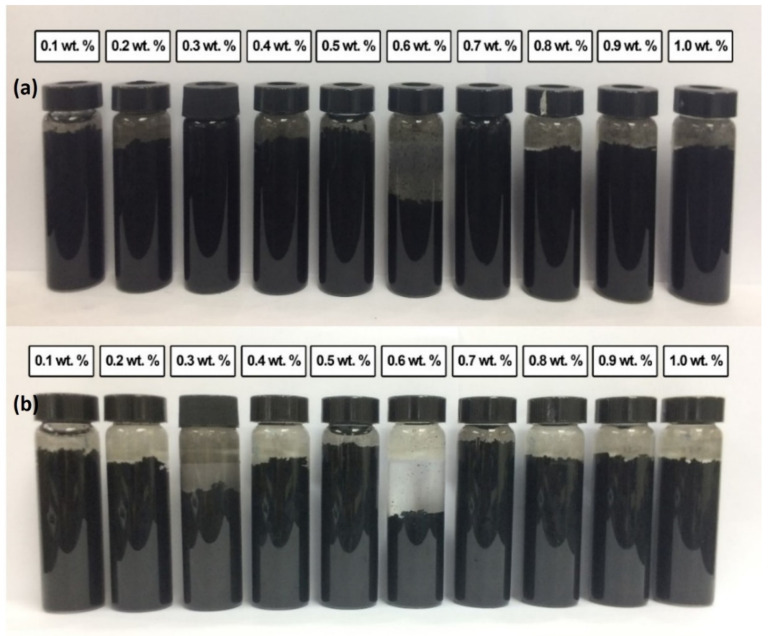
Stability and dispersion condition of various weight percentages of the commercial MWCNTs based nanofluid without surfactant for (**a**) 0.5 h; (**b**) 100 h after homogenization and sonication process.

The nanofluids are considered stable when there is no particle sedimentation. The sedimentation of each sample was captured at two different periods, which is 0.5 h after the homogenization and sonication process, and the second is after 100 h. Producing a stable nanofluid becomes more challenging for suspensions containing carbon particles. MWCNTs are hydrophobic and usually exist in bundles, and at the same time, MWCNTs have a very high aspect ratio, making them easy to agglomerate into different sizes and shapes, but the graphite powder directly forms sediment. This means that when MWCNTs nanoparticles are dispersed in the base fluid, the agglomeration and sedimentation occur quickly, and the suspensions are unstable. Figure 5a,b shows the condition of various weight percentages of the commercial MWCNTs-based nanofluid (0.1 wt.%, 0.2 wt.%, 0.3 wt.%, 0.4 wt.%, 0.5 wt.%, 0.6 wt.%, 0.7 wt.%, 0.8 wt.%, 0.9 wt.% and 1.0 wt.%) without surfactant for 0.5 h and 100 h after the homogenization and sonification process. Visual rapid sedimentation and coagulation were observed 0.5 h after homogenization and sonification, except for 0.3 wt.% and 0.7 wt.%. According to Phuoc et al. (2011), carbon nanotube-based nanofluid without surfactant quickly disappeared after the ultrasonication process [48]. After 100 h, it can be clearly seen that sedimentation occurred to all the samples of commercial MWCNTs based nanofluids, and the amounts of sedimentation are different. All the weight concentrations of commercial MWCNTs are considered unstable, but the 0.5 wt.% and 0.7 wt.% have fewer particles settling down compared to others. This phenomenon is attributed to the highly hydrophobic surface of MWCNTs itself. Van der Waal’s attractive force is stronger; consequently, the MWCNTs tend to attract each other. This is consistent with our FESEM images. Additionally, the homogenization and sonication processes alone are not enough to produce a stable nanofluid sample. According to Ali et al. (2019), unstable nanofluids form three behaviours, namely dispersed sedimentation, flocculated sedimentation, and mixed sedimentation [49]. In this work, rapid dispersion of the sedimentation occurs after 0.5 h due to the separation of MWCNTs from water. While, after 100 h flocculated sedimentation was observed. A decrease in stability along the shelving period time is the result of weak physical interaction of MWCNTs with the base fluids followed by separation of the particles from the water and form sedimentation.

**Figure 6:**
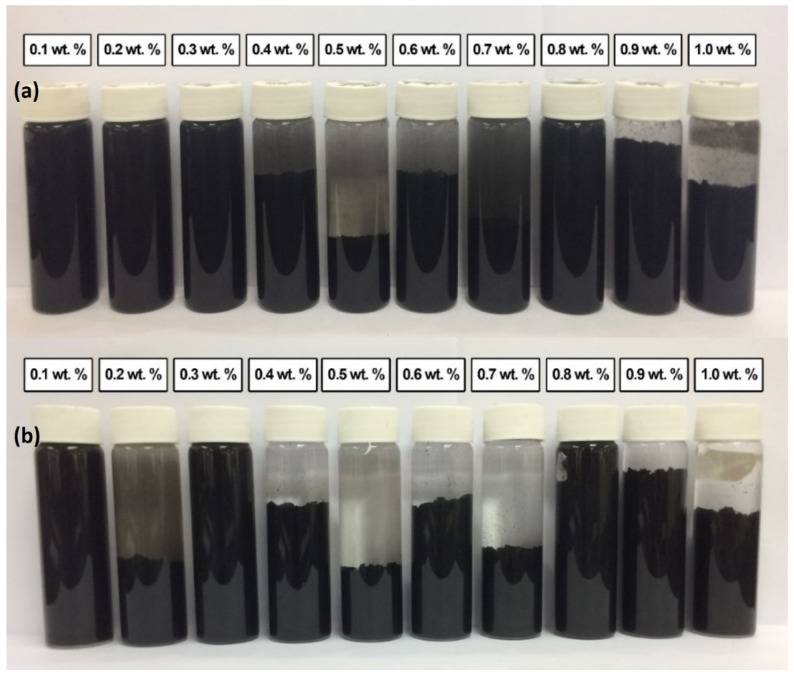
Stability and dispersion condition weight percentage of the commercial MWCNTs based nanofluid with surfactant of PVP for (**a**) 0.5 h; (**b**) 100 h after homogenization and sonication process.

Figure 6a,b shows the condition of various weight percentages of the commercial MWCNTs based nanofluid with a surfactant of PVP. Adding surfactant will adjust the hydrophobicity nature of MWCNTs into a hydrophilic surface that might prevent agglomeration. As can be seen, the dispersion stability improved slightly compared to nanofluids without PVP with similar settling sedimentation behaviour. Nanofluids contain a low concentration of MWCNTs show stability with no sedimentation. However, rapidly dispersed sedimentation occurred in the samples at 0.4 wt.%, 0.5 wt.%, 0.6 wt.% 0.7 wt.%, 0.9 wt.% and 1.0 wt.% after 0.5 h. This sedimentation continuously occurred at 0.2 wt.% of commercial MWCNTs based nanofluid after 100 h. The PVP is added to reduce the surface tension between solid and liquid, thus improving the dispersing of particles and preventing the particles from settling. However, in this experimentation, particle coagulation and sedimentation occurred. This result can be due to the weight percentage of PVP, which is a 10% concentration of carbon particle, which is not suitable for a certain weight percentage of MWCNTs and made the nanofluids unstable. The duration of homogenization and sonication process that is too long also can cause sedimentation to occur [48]. However, no sedimentation was observed for 0.1 wt.% and 0.3 wt.% of commercial MWCNTs based nanofluids after 100 h. On the other hand, the introduction of nonionic surfactant PVP in the formulation has less influence on the stability of the as-prepared nanofluids.

**Figure 7:**
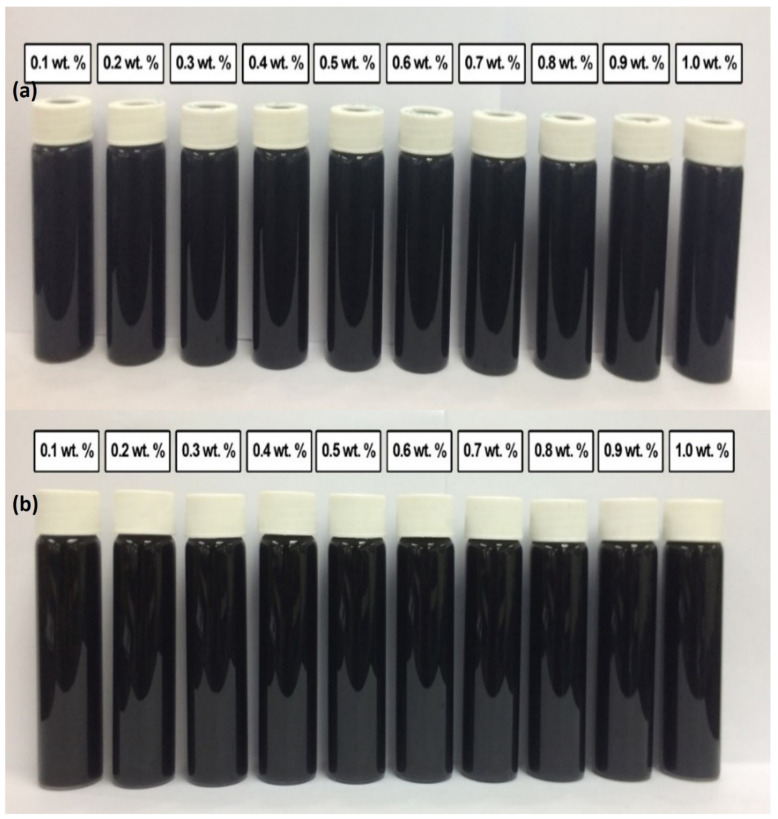
Stability and dispersion condition of various weight percentage of the surface oxidized MWCNTs (MWCNT-MB) based nanofluid without surfactant for (**a**) 0.5 h; (**b**) 100 h after homogenization and sonication process.

Figure 7 shows the stability and dispersion condition of various weight percentages of the surface oxidized MWCNTs without surfactant for (a) 0.5 h; (b) 100 h after the homogenization and sonication process. No sedimentation occurred in surface oxidized MWCNTs nanofluids, but only a small amount of sedimentation after 100 h. Therefore, all samples are considered stable. Stable nanofluids were due to functional groups (-COOH and O-H) on the surface walled of MWCNTs. The presence of SOFG led to a reduction of van der Waals interaction among them, which promotes their separation and dispersion in the nanofluids compared with commercial MWCNTs nanoparticles. On the other hand, the sonication technique applied during the surface oxidation, as well as in the dispersion process, also had an energetic effect in getting the MWCNTs bundles to start to loosen [50,51].

The same situation also occurred in the condition of various weight percentages of the surface oxidized MWCNTs (MWCNT-MB) based nanofluid with a surfactant of PVP for (a) 0.5 h; (b) 100 h after homogenization and sonication process. No sedimentation occurred in surface oxidized MWCNTs samples. The presence of SOFG in the nanofluids led the MWCNTs to be dispersed well. The presence of surfactant PVP improved the stability of the nanofluids in this experimental study. As known, the production of a nanofluid faces some significant challenges, such as agglomeration of nanoparticles in solution and the rapid settling of nanoparticles in fluids, which will cause settlement that clogs flow channels and degenerates the fluid’s overall effectiveness. Some factors contribute to the stability and dispersion of carbon-based nanofluids, such as hydrophobic nanocarbon in nature, the addition of surfactant, surface modification of nanocarbon, and homogenization and ultrasonication process. The hydrophobic of nanocarbon against fluid makes it possible to form agglomerates. In this study, the commercial MWCNTs nanofluid without PVP showed sedimentation occurred in all samples after 100 h of observation. After surface modification, nanofluid containing surface oxidized MWCNTs without PVP showed no sedimentation in the samples on the bottom of the bottle. However, all these samples are considered stable. It proved that the surface modification process of nanocarbon could improve the stability of nanofluids. A significant number of oxygen-containing functionalities at the defect sites (evidence from Raman spectrum and FESEM images) contribute to the acid-treated MWCNT’s stability in polar fluids.

Furthermore, the addition of PVP helps to improve the stability of nanofluids. The result of stability of commercial MWCNTs based nanofluid with PVP is better compared to without PVP. Furthermore, the addition of PVP improved the stability of nanofluids and prevented their settlement in water without undergoing acid treatment. After acid treatment, the surface oxidized nanocarbon-based nanofluid containing PVP showed better stability. This stability is also much better than surface oxidized nanocarbon-based nanofluids without PVP. In general, the nanofluids prepared using MWCNT-B have demonstrated better and long-term stability than those produced using commercial MWCNTs.

### 3.6. Thermal Conductivity of Commercial MWCNTs Based Nanofluids

**Figure 8:**
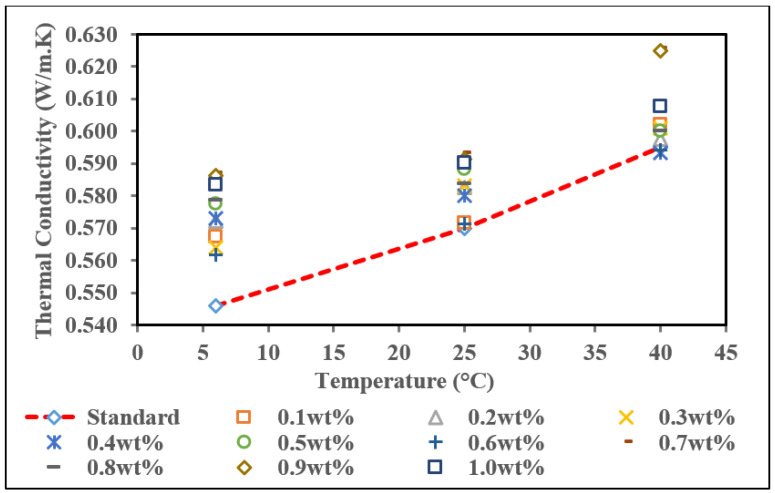
Thermal conductivity of commercial MWCNTs based nanofluid without PVP with various concentration at different temperatures.

The result of thermal conductivity of commercial MWCNTs nanofluid without PVP at three different temperatures is represented in Figure 8 to identify the patterns of the heat effects on each sample. The thermal conductivity for pure base fluid (standard) was 0.546 W/m·K, 0.570 W/m·K, and 0.595 W/m·K at 6 °C, 25 °C, and 40 °C, respectively, and presented in a straight line in Figure 8. These thermal conductivity values are standard values for comparison with the thermal conductivity of commercial MWCNTs based nanofluid without PVP. Based on the graph, the trend for this nanofluid is increasing thermal conductivity with increasing temperature. The thermal conductivity values are above the pure base fluid (standard) as the temperature increases from 6 °C to 25 °C. However, at a temperature of 40 °C, few samples record thermal conductivity lower than the standard value, which is at weight concentrations of 0.3 wt.% and 0.4 wt.%. In comparison, the other samples continuously increase as the temperature increases to 40 °C.

The highest thermal conductivity at all temperatures was recorded at 0.7 wt.%. The highest reading for the thermal conductivity was 0.626 W/m·K at 40 °C, followed by 0.593 W/m·K at 25 °C and 0.587 W/m·K at 6 °C. This result corresponds well with the theory that thermal conductivity increase is a function of increased Brownian movement between MWCNTs particles and deionized water particles [52]. However, for the lowest thermal conductivity, it happens at a concentration of 0.6 wt.% of commercial MWCNTs nanofluid for 6 °C and 25 °C, which is 0.562 W/m·K and 0.571 W/m·K. At 40 °C, 0.4 wt.% of commercial MWCNTs nanofluid has the lowest thermal conductivity value among the other concentrations. These findings are similar to Jiang et al. (2015) on CNT water-based nanofluid research, which reported that the thermal conductivity value increases nonlinearly as the volume fraction increases [53]. The thermal conductivity enhancement (*K_enhancement_*) analysis compared to the pure base fluid with the nanofluids sample is shown in Table 5. The percentage enhancement was calculated according to Equation (2), where *K_nf_* and *K_f_* represent the thermal conductivity of the nanofluid and the pure base fluid, respectively.

(2) $$K_{enhancement\;}\left(\%\right)=\left[\frac{K_{nf}-K_{f}}{K_{f}}\right]\times 100\;\%$$

Referring to Table 5 for thermal conductivity enhancement of commercial MWCNTs nanofluid without PVP, an irregular trend was observed in terms of the enhancement at all temperatures. At 0.3 wt.% and 0.4 wt.% weight concentration, there was no enhancement in the thermal conductivity of nanofluid concerning the pure base fluid. These anomalous results of the thermal conductivity value are due to the clustering phenomenon in agreement with [54,55]; hence, the nanoparticles in these samples might have higher agglomeration/cluster dimension, which can alter the thermal conductivity measurement compared to others. This is in line with our stability analysis; both nanofluids give rapid settlement of sedimentation after 100 h shelving time. It is noted that well-dispersed nanoparticles are a crucial factor in improving the thermal conductivity of nanofluids. Most of the nanofluid samples exceed the pure base fluid in thermal conductivity enhancement and the highest percentage enhancement recorded is at a temperature of 40 °C, which is 10.25%. This enhancement is due to the intrinsic heat transport capacity of the MWCNTs [56]. Overall, a concentration of 0.7 wt.% is the optimum weight concentration for commercial MWCNTs based fluid and is in line with our stability test observation. According to Jang et al. (2004), temperature changes directly influence Brownian motion, impacting nanoparticle behaviours such as particle collisions, thermal interactions, and diffusion. As the temperature is increased, the viscosity of base fluids is also decreased, the Brownian motion of nanoparticles is increased, and consequently, convection-like effects are remarkably increased, resulting in increased conductivities [57].

**Figure 9:**
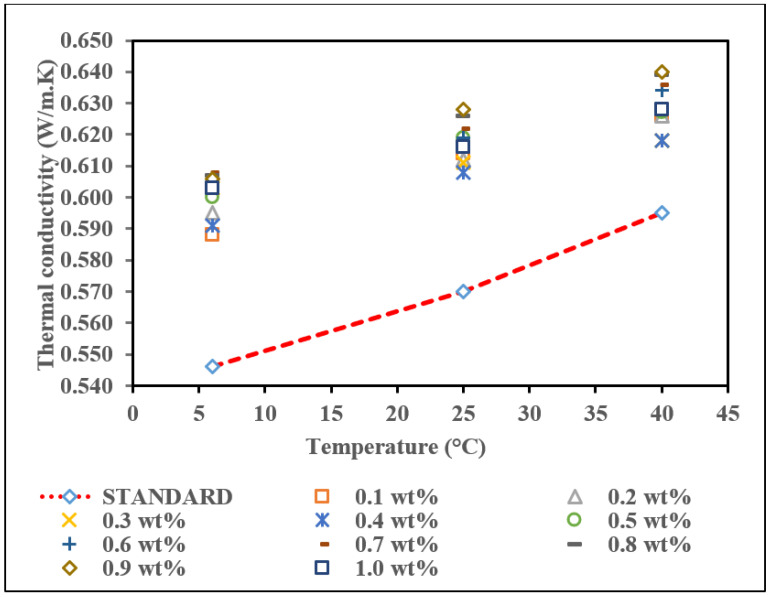
Thermal conductivity of commercial MWCNTs based nanofluid with PVP with various concentrations at different temperatures.

As shown in Figure 9, the results varied for commercial MWCNTs based nanofluid with a surfactant to increase thermal conductivity. By adding 10% of PVP, the thermal conductivity of all weight concentrations of commercial MWCNTs increased linearly with the temperature rise. The same trend also happened to the thermal conductivity value increases as the concentration of MWCNTs increased. 

Adding PVP prevents the clustering and coagulation of nanofluid from occurring and improves the thermal conductivity. The thermal conductivity of nanofluids depends on temperature, as shown in Figure 9. The thermal conductivity of each nanofluid increases with increasing temperature, with the highest thermal conductivity being at a temperature of 40 °C, followed by 25 °C and 6 °C. Based on Figure 9, the highest thermal conductivity at all temperatures was recorded for 0.9 wt.%. The highest reading for the thermal conductivity at temperature 6 °C was gained at 0.608 W/m·K, whereas at 25 °C, the highest thermal conductivity reading was 0.628 W/m·K, followed by 0.650 W/m·K at 40 °C. The lowest thermal conductivity reading at 6 °C is 0.598 W/m·K at a concentration of 0.1 wt.%, and at 25 °C, the lowest thermal conductivity is 0.608 W/m·K at a concentration of 0.4 wt.%. At 40 °C, two concentrations have similar values (0.618 W/m·K), which are 0.3 wt.% and 0.4 wt.%. In this study, the thermal conductivity of nanofluid did not depend on the weight concentration of commercial MWCNTs and PVP. However, the reading of thermal conductivity was above the pure base fluid, and the enhancement is shown in Table 6.

The highest percentage enhancements for MWCNTs nanofluid with PVP at 6 °C, 25 °C, and 40 °C were obtained at 0.9 wt.%, which is the optimum concentration for this nanofluid. This concentration’s thermal conductivity percentage enhancement was 11.36%, 10.23%, and 7.62% at 6 °C, 25 °C, and 40 °C, respectively. However, the concentration of 0.3 wt.% at 40 °C is the lowest enhancement, 3.81%. Although the enhancement is the lowest, at least the presence of MWCNTs in the base fluid has enhanced the thermal conductivity rather than the thermal conductivity of the standard. In addition, at a concentration of 1.0 wt.%, the result is declined where the enhancement of thermal conductivity is 10.50%, 8.07%, and 4.65% at 6 °C, 25 °C, and 40 °C, respectively. Generally, the thermal conductivity increased with increasing weight concentration. In this study, the increase in the weight concentration of commercial MWCNTs based nanofluid did not significantly increase the thermal conductivity. These results are supported by Ravi et al. (2013), who found that the nonlinear relationship between nanofluids has low and fluctuating thermal conductivity with increasing concentration. The size and shape of MWCNTs may be the main factor in the nonlinear relationship between thermal conductivity and MWCNTs loading. The results show that MWCNTs interact with each other due to their high aspect ratio, even with low MWCNTs loading [58]. Overall, the thermal conductivity enhancement of the commercial MWCNTs nanofluids with surfactant is higher than without surfactant [59].

### 3.7. Thermal Conductivity of Surface Oxidized MWCNTs Based Nanofluids (MWCNT-MB)

**Figure 10:**
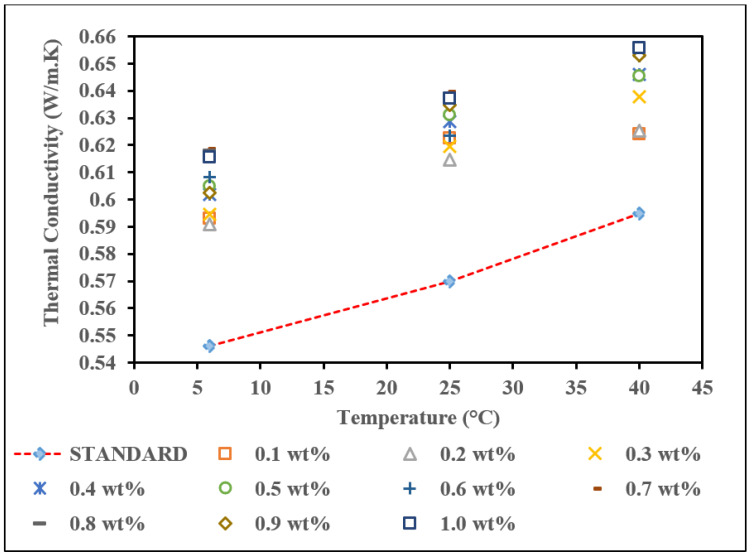
Thermal conductivity of surface oxidized MWCNTs based nanofluid without PVP with various concentrations at different temperatures.

**Figure 11:**
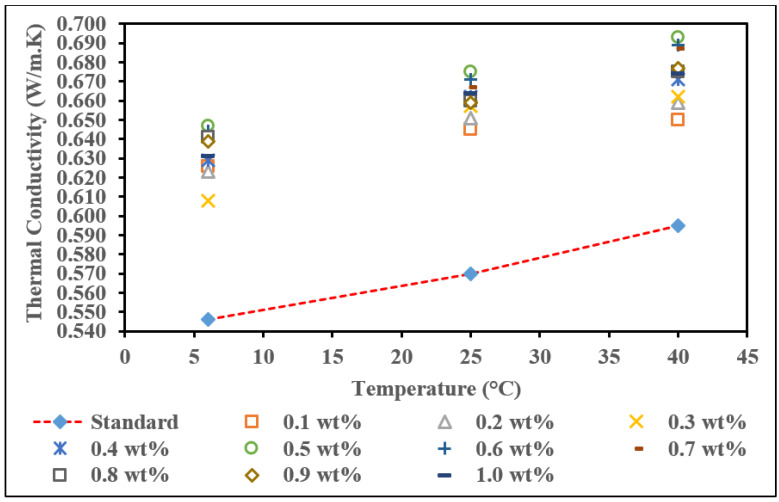
Thermal conductivity of base fluid containing surface oxidized MWCNTs with PVP with various concentrations at different temperatures.

Thermal conductivity of surface oxidized MWCNTs based nanofluids results with and without surfactant, PVP at different temperatures is presented in Figure 10 and Figure 11, respectively. Figure 10 shows the thermal conductivity of the base nanofluid containing surface oxidized MWCNTs clearly shows that the thermal conductivity of base fluid containing surface oxidized MWCNTs had higher thermal conductivity than pure base fluid and on the thermal conductivity of commercial MWCNTs based fluids. The increments of both properties are about two times that of the commercial-based nanofluids. In Figure 10, it was observed that thermal conductivity enhancements of ultrapure water-based nanofluids containing surface oxidized MWCNTs showed augmentation for temperature for each concentration MWCNTs. Based on Table 7, the weight concentration of surface oxidized MWCNTs is not significant due to the fluctuation in increment of thermal conductivity. The thermal conductivity shows increment until the concentration of 0.7 wt.%, the decrement starts at a concentration of 0.8 wt.% to 1.0 wt.%. This is due to when the weight concentration is beyond 0.5 wt.%, and surface oxidized MWCNTs tend to agglomerate and reduce thermal conductivity performance. However, as observed, the temperature is more critical due to the thermal conductivity of all weight concentrations increasing when temperature increases. This is because the Brownian motion of nanoparticles and the -OH group on the MWCNTs surface tend to transfer more energy to nanofluids through increments in temperature [60,61]. In addition, surface modification or functionalization may also lead to more substantial thermal conductivity enhancements. The functionalized CNTs move fast in the water so that energy transport inside the liquid becomes strong and thermal conductivity increases [62]. While, at 6 °C, 25 °C, and 40 °C, the highest thermal conductivity was at a concentration of 0.7 wt.%, where the values were 0.619 W/m·K, 0.640 W/m·K, and 0.657 W/m·K, respectively. Meanwhile, the lowest thermal conductivity happens at a concentration of 0.1 wt.% at 6 °C and 40 °C, which is 0.583 W/m·K, and 0.614 W/m·K.

At 25 °C, 0.3 wt.% of surface oxidized MWCNTs concentration has the lowest thermal conductivity value compared to the other concentrations. Although these nanofluid samples had the lowest thermal conductivities, these values were still enhanced as compared to the pure-base fluid, where there was an enhancement of 8.61%, 8.71%, and 4.87% (Table 5). The highest percentage of enhancement for each temperature is 13.31% at a temperature of 40 °C, 12.22% at a temperature of 25 °C, and 10.42% at a temperature of 6 °C. This enhancement is at a concentration of 0.7 wt.% of surface oxidized MWCNTs, which is the optimum weight concentration for this nanofluid.

Figure 11 represents the thermal conductivity of base fluid containing surface oxidized MWCNTs with surfactant. Based on the value of percentage enhancement, surface oxidized MWCNTs nanofluid had a higher percentage compared to commercial MWCNTs. This is due to the surface oxidation MWCNTs that have a very small particle size. Theoretical evidence indicates that the effect of thermal conductivity of nanofluid increased with decreased particle size [63]. Additionally, -OH groups on the surface of MWCNTs tend to transfer more energy to the nanofluid through temperature increase. From the results in Figure 11 it could be seen that the increase in thermal conductivity varies with an increase in the weight percentage of nanoparticles and starts to decrement at a concentration of 0.7 wt.% to 1.0 wt.%. The decrement is due to the increase in the weight concentration that caused an increase in the viscosity of the nanofluid and may have affected the Brownian motion of the suspended nanoparticles [64]. Additionally, the decrement happened due to the side effect of surfactant [33]. The highest thermal conductivity at all temperatures was recorded at a concentration of 0.5 wt.%. The value of thermal conductivity is 0.647 W/m·K at 6 °C, 0.675 W/m·K at 25 °C and 0.693 W/m·K at 40 °C, respectively. It shows that the thermal conductivity increases with increasing temperature.

The enhancement analysis was conducted to observe the trend and is shown in Table 8 by the irregular enhancements in thermal conductivity. The enhancement of thermal conductivity shows increment until at concentration of 0.5 wt.% and then the decrement as started at a concentration of 0.6 wt.% to 1.0 wt.%. This is due to when weight concentrations go beyond 0.5 wt.% surface oxidized MWCNTs tend to agglomerate and reduce the performance of thermal conductivity. However, the enhancement was improved compared to the surface oxidized MWCNTs without PVP. It proved that the addition of surfactant improved the thermal conductivity of nanofluid. Based on Table 6, the nanofluid sample with a concentration of 0.5 wt.% had the highest enhancement in thermal conductivity of 18.50%, 18.42%, and 16.47% at 6 °C, 25 °C, and 40 °C, respectively. Two different concentrations show the lowest enhancement of thermal conductivity at all temperatures, which are 0.3 wt.% at 6 °C, and 0.1 wt.% at 25 °C, and 40 °C.

The present study shows that the mobility of nanoparticles, which includes temperature dependency and particles stability, is the dominating factor for the thermal conductivity enhancement of nanofluids more than any other factor. Therefore, it can be stated that at a higher temperature, the nanofluid thermal conductivity increases primarily due to increasing nanoparticle Brownian motion [65]. Whereas the formulation of surface functional groups of MWCNT blended together with non-ionic surfactant is the main factor for improving thermal conductivity.

## 4. Conclusions

MWCNTs have been successfully chemically modified using the acid treatment method to introduce the SOFG on the MWCNTs wall structure. FESEM revealed that the mean diameter of commercial MWCNTs increased from 26 nm to 38 nm for method A and 43 nm for method B, respectively. It was unable to determine the diameter length of MWCNTs produced from method C due to the damaged structure, as confirmed by FESEM observation. The Raman spectroscopy proved the presence of SOFG on MWCNTs by showing a greater ID/IG ratio due to the presence of functional groups attached to the MWCNTs was confirmed by TGA and FTIR spectroscopy. TGA showed additional weight losses due to the elimination of oxygenated functional groups. FTIR spectrum confirmed the attachment of oxygenated functional groups by appearing essential bands such as C=O (from carboxyl group), O-H, C=O, and C-O. The surface oxidized MWCNT with PVP in water-based nanofluids is more stable than non-oxidized MWCNTs with PVP and commercial MWCNTs with and without PVP by the observation on the particle sedimentation and coagulation. The thermal conductivity performance of nanofluids revealed that the surface oxidized MWCNTs with PVP shows enhancement in thermal conductivity contributed by improved stability and homogenization of nanoparticles. Hence improved the distribution of MWCNTs in water base media leads to improvement in thermal conductivity. These promising properties of MWCNTs in water-based fluids would enable the nanofluids to be used in heat transfer fluid and cooling applications.

## Figures and Tables

**Table 1:** Summary of three (3) acid treatment methods.

Method	Sample Code	Ultrasonication Process		Reflux Process	
		Times	Temperature (K)	Times	Temperature (K)
A	MWCNT-MA	30 min	313	180 min	423
B	MWCNT-MB	2 h	343	-	-
C	MWCNT-MC	6 h	343	-	-

**Table 2:** Nanofluids formulation using commercial and modified MWCNTs with PVP.

Weight Conc. of MWCNTs (wt.%)	Mass of MWCNTs (g)	Weight Conc. of PVP (wt.%)	Mass of PVP (g)	Volume of Deionized Water (mL)	Total Volume of n Nanofluid (mL)
0.1	0.04	0.01	0.004	39.96	40.00
0.2	0.08	0.02	0.008	39.91	40.00
0.3	0.12	0.03	0.012	39.87	40.00
0.4	0.16	0.04	0.016	39.82	40.00
0.5	0.20	0.05	0.020	39.78	40.00
0.6	0.24	0.06	0.024	39.74	40.00
0.7	0.28	0.07	0.28	39.69	40.00
0.8	0.32	0.08	0.032	39.65	40.00
0.9	0.36	0.09	0.036	39.60	40.00
1.0	0.40	0.10	0.040	39.56	40.00

**Table 3:** Nanofluids formulation using commercial and modified MWCNTs without PVP.

Weight Conc. of MWCNTs (wt.%)	Mass of MWCNTs (g)	Weight Conc. of PVP (wt.%)	Mass of PVP (g)	Volume of Deionized Water (mL)	Total Volume of Nanofluid (mL)
0.1	0.04	-	-	39.96	40.00
0.2	0.08	-	-	39.92	40.00
0.3	0.12	-	-	39.88	40.00
0.4	0.16	-	-	39.84	40.00
0.5	0.20	-	-	39.80	40.00
0.6	0.24	-	-	39.76	40.00
0.7	0.28	-	-	39.72	40.00
0.8	0.32	-	-	39.68	40.00
0.9	0.36	-	-	39.64	40.00
1.0	0.40	-	-	39.60	40.00

**Table 4:** The value of I_D_/I_G_ ratio for commercial and surface oxidation of MWCNTs.

Sample	I_D_/I_G_
Commercial MWCNTs	0.75
MWCNT-MA	0.87
MWCNT-MB	0.91
MWCNT-MC	0.82

**Table 5:** Thermal conductivity enhancement of commercial MWCNTs nanofluid without PVP.

Weight Concentration of Commercial MWCNTs (wt.%)	Percentage of *K_enhancement_* (%) at Different Temperature		
	6 °C	25 °C	40 °C
0.1	3.91 (0.05)	0.29 (0.06)	1.23 (0.06)
0.2	4.82 (0.04)	2.16 (0.06)	0.45 (0.07)
0.3	3.30 (0.04)	0.25 (0.03)	-
0.4	4.95 (0.06)	1.75 (0.04)	-
0.5	5.80 (0.07)	3.22 (0.07)	0.90 (0.05)
0.6	2.87 (0.07)	2.28 (0.07)	1.01 (0.1)
0.7	7.51 (0.06)	4.09 (0.09)	10.25 (0.09)
0.8	5.98 (0.1)	2.40 (0.09)	0.90 (0.05)
0.9	7.39 (0.9)	3.74 (0.2)	5.60 (0.09)
1.0	6.84 (0.06)	3.57 (0.1)	2.13 (0.1)

(error bar).

**Table 6:** Thermal conductivity enhancement of commercial MWCNTs nanofluid with PVP.

Weight Concentration of Commercial MWCNT s (wt.%)	Weight Concentration of PVP (wt.%)	Percentage of *K_enhancement_* (%) at Different Temperature		
		6 °C	25 °C	40 °C
0.1	0.01	7.63 (0.01)	7.78 (0.02)	5.27 (0.04)
0.2	0.02	8.97 (0.04)	7.43 (0.03)	5.21 (0.03)
0.3	0.03	10.38 (0.02)	7.13 (0.05)	3.81 (0.009)
0.4	0.04	8.24 (0.009)	6.67 (0.04)	3.92 (0.04)
0.5	0.05	9.89 (0.01)	8.65 (0.06)	5.38 (0.03)
0.6	0.06	10.44 (0.03)	8.54 (0.05)	6.55 (0.01)
0.7	0.07	10.99 (0.01)	9.12 (0.02)	6.89 (0.04)
0.8	0.08	11.17 (0.05)	9.82 (0.03)	7.34 (0.009)
0.9	0.09	11.36 (0.06)	10.23 (0.07)	7.62 (0.02)
1.0	0.10	10.50 (0.06)	8.07 (0.07)	4.65 (0.02)

(error bar).

**Table 7:** Thermal conductivity enhancement of surface oxidized MWCNTs nanofluid without PVP.

Weight Concentration of Surface Oxidized MWCNTs (wt.%)	Percentage of *K_enhancement_* (%) at Different Temperature		
	6 °C	25 °C	40 °C
0.1	8.61 (0.003)	9.24 (0.002)	4.87 (0.001)
0.2	10.07 (0.006)	9.59 (0.006)	6.78 (0.005)
0.3	8.91 (0.005)	8.71 (0.005)	7.17 (0.004)
0.4	10.26 (0.004)	10.29 (0.007)	8.57 (0.006)
0.5	10.81 (0.006)	10.70 (0.005)	8.46 (0.009)
0.6	11.42 (0.003)	9.36 (0.005)	9.69 (0.008)
0.7	13.31 (0.002)	12.22 (0.006)	10.42 (0.001)
0.8	13.19 (0.01)	11.99 (0.01)	10.25 (0.005)
0.9	10.32 (0.008)	11.35 (0.02)	9.75 (0.004)
1.0	12.76 (0.01)	11.81 (0.007)	10.20 (0.007)

(error bar).

**Table 8:** Thermal conductivity enhancement of surface oxidized MWCNTs nanofluid with PVP.

Weight Concentration of Surface Oxidized MWCNT s (wt.%)	Weight Concentration of PVP (wt.%)	Percentage of *K_enhancement_* (%) at Different Temperature		
		6 °C	25 °C	40 °C
0.1	0.01	14.65 (0.001)	13.16 (0.002)	9.24 (0.002)
0.2	0.02	14.10 (0.003)	14.21 (0.001)	10.76 (0.003)
0.3	0.03	11.36 (0.002)	15.26 (0.002)	11.26 (0.006)
0.4	0.04	15.20 (0.001)	16.14 (0.003)	12.77 (0.001)
0.5	0.05	18.50 (0.004)	18.42 (0.004)	16.47 (0.002)
0.6	0.06	17.95 (0.005)	17.72 (0.001)	15.80 (0.004)
0.7	0.07	17.22 (0.003)	17.02 (0.002)	15.46 (0.002)
0.8	0.08	17.40 (0.003)	15.79 (0.001)	13.45 (0.003)
0.9	0.09	17.03 (0.004)	15.61 (0.003)	13.78 (0.004)
1.0	0.10	15.57 (0.004)	16.49 (0.003)	13.28 (0.008)

(error bar).

## Data Availability

The data is available on reasonable request from the corresponding author.

## Nomenclature

Symbol	Description, Unit
I_D_/I_G_	Intensity ratio, -
*K_f_*	Thermal conductivity of the fluid, W/m·K
*K_nf_*	Thermal conductivity of the nanofluid, W/m·K
*K_enhancement_*	Thermal conductivity enhancement, %
